# Prosthetic-joint Infections: Mortality Over The Last 10 Years

**DOI:** 10.7150/jbji.35428

**Published:** 2019-09-17

**Authors:** Arnaud Fischbacher, Olivier Borens

**Affiliations:** Centre hospitalier universitaire vaudois (CHUV), Service d'orthopédie et de traumatologie, Rue du Bugnon 46, 1011 Lausanne, Switzerland.

**Keywords:** prosthetic-joint infection, mortality

## Abstract

**Background:** There is a constant increase of joint arthroplasties to improve the quality of life of an ever-aging population. Although prosthetic-joint infections are rare, with an incidence of 1-2%, they represent a serious complication in terms of morbidity and mortality. Infection related mortality is known to be approaching 8% at one year. The aim of this retrospective study is to reassess the one and two-year mortality over the last ten years.

**Methods:** Patients treated for prosthetic joint infection at the University Hospital of Lausanne (Switzerland) between 2006 and 2016 were included. The one and two-year cumulative mortality depending on sex, age, type of prosthesis, infecting organism and type of surgical treatment were computed.

**Results:** 363 patients (60% hips, 40% knees) were identified with a median age of 70 years. The one-year cumulative mortality was 5.5% and it was 7.3% after two years. No difference was seen between hip and knee prostheses, but the mortality was higher in men than in women and increased with age. Furthermore, there was a significant difference depending of the germ with enterococci infections associated with a higher risk of death. Finally, patients treated with a one-stage or two-stage exchange had a lower mortality than those treated with debridement and retention.

**Conclusion:** The mortality is still high and differs according to sex, age, infecting organism and type of surgical treatment. There is a need of studies to improve the management of patients at risk of increased mortality.

## Introduction

In Switzerland, more than 35'000 joint arthroplasty procedures are performed every year to improve the quality of life of an ever-aging population.^1^ Prosthetic-joint infections (PJI) are rare, with an incidence of 1-2%, but they represent serious complications in terms of morbidity and costs.^2^ Furthermore, a significant number of them lead to fatal outcomes. The disease process of prosthetic-joint infections itself, the need for aggressive surgical treatments and the long-term use of systemic antibiotics may alter life expectancy.^3^-^6^ A study by Overgaard et al. showed that 8% of the patients treated for prosthetic-joint infections after total hip arthroplasty died within 1 year compared to 3% in those who did not undergo revision for any cause.^7^ Zmistowski et al. even compared the relative survival rate at five years of prosthetic joint infections, which was 87.3%, to the one of the top five most common cancers which are prostate cancer (99%), breast cancer (89%), lung cancer (16%), colorectal cancer (64%) and melanoma (91%).^3^

Given such a devastating complication, this retrospective study intends to reassess the one and two-year mortality over the last ten years depending on sex, age, type of prosthesis, infecting organism and type of surgical treatment.

## Methods

**Figure 1:**
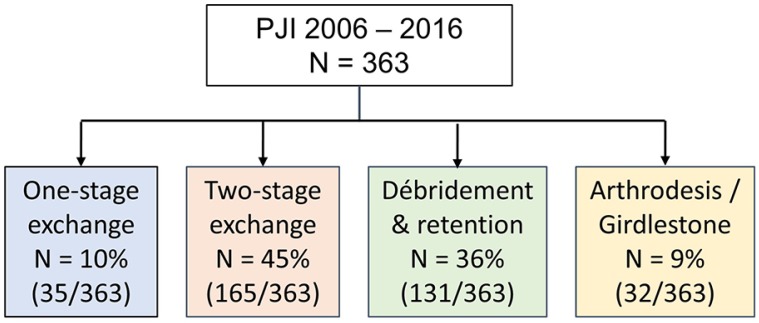
Flow of the patient selection.

The inclusion criterion was to have a prosthetic-joint infection, as defined by the Infectious Diseases Society of America, which was surgically treated at the University Hospital of Lausanne (CHUV, Switzerland) from January 2006 to December 2016 [Figure 1].^8^ Data for the included patients were extracted from electronic files and retrospectively analyzed. Each patient's vital status, as at 31 December 2018, was identified through our institutional medical records. If the patient had died, the date of death was recorded. Cumulative mortality (proportion of patients alive at the start of a period that die over that period) within one year and two years of the prosthetic-joint infection treatment was estimated using Kaplan-Meier analysis with a log rank test and a Cox's regression to determine if there were differences between groups.

## Results

### Patients

363 prosthetic-joint infections were treated in the Department of Septic Surgery at the University of Lausanne from 2006 to 2016 [Table 1]. 189 patients were followed up to one year and 102 up to 2 years. The median follow-up was 14.4 months.

### Cumulative mortality

**Figure 2:**
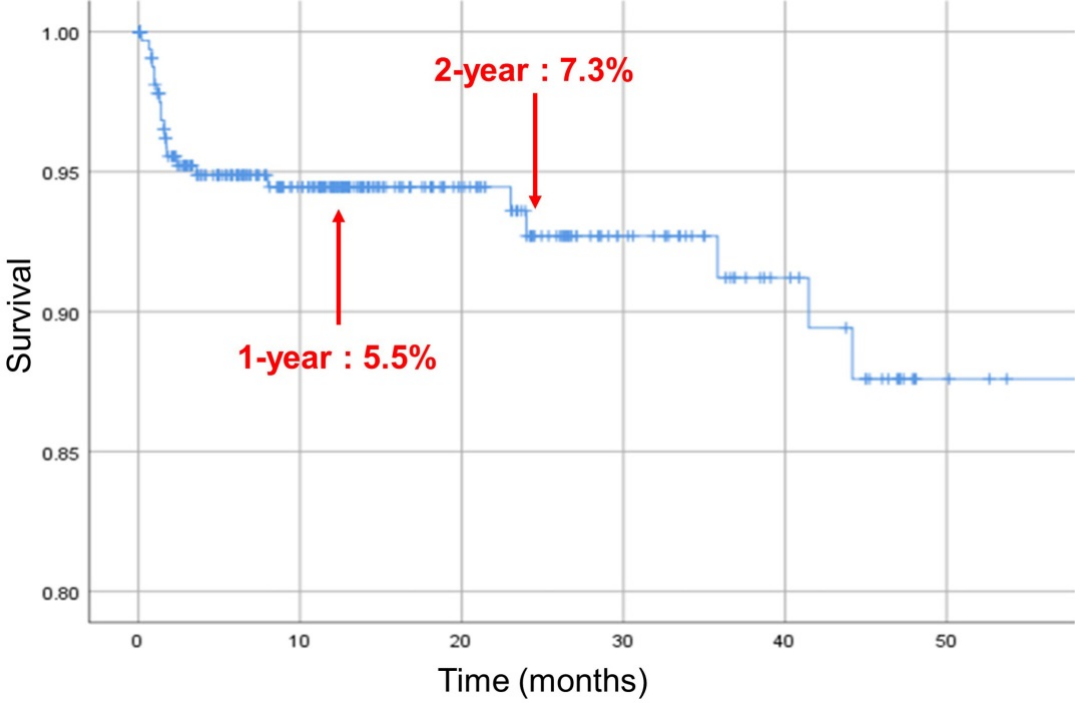
Kaplan-Meier survival curve. Vertical marks indicate censoring.

In this cohort, 17 patients died during the first year and 2 during the second year. The cumulative mortality was 5.5% within one year and 7.3% within two years [Figure 2].

### Mortality depending on sex

**Figure 3:**
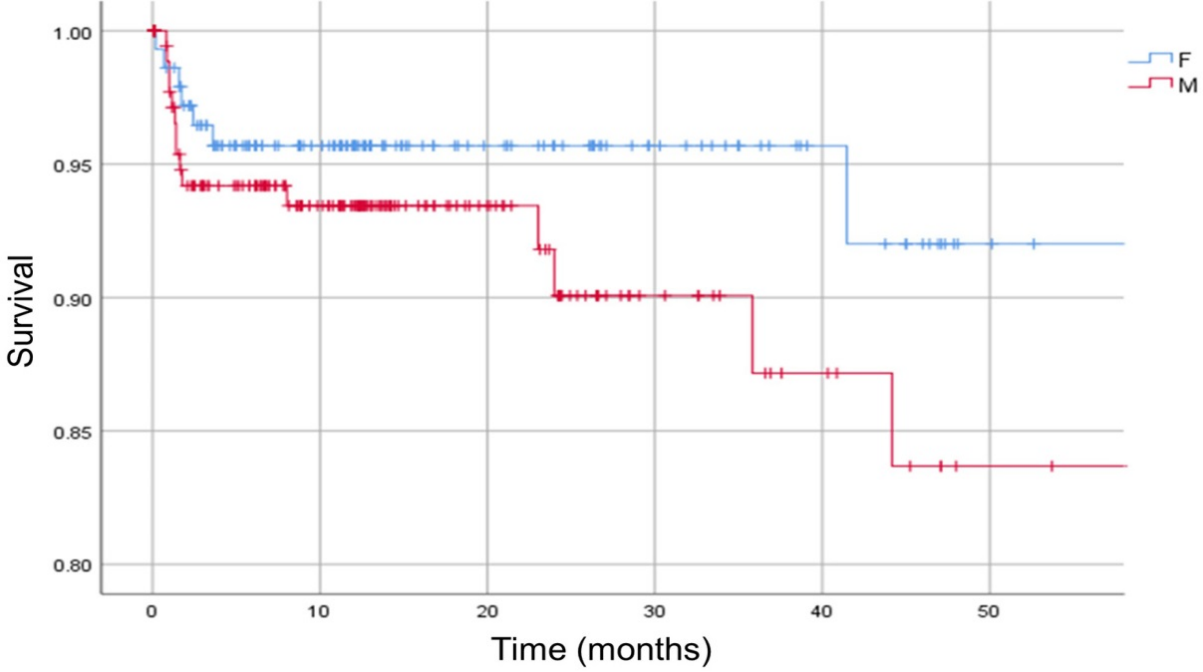
Kaplan-Meier survival curve. The analysis is separated in females (F) and males (M). Vertical marks indicate censoring.

Male patients undergoing revision for prosthetic-joint infection were more likely to die within 1 and 2 years than female patients (p < 0.05, HR 2.00, 95% CI 0.88-4.57) [Figure 3].

### Mortality depending on age

**Figure 4:**
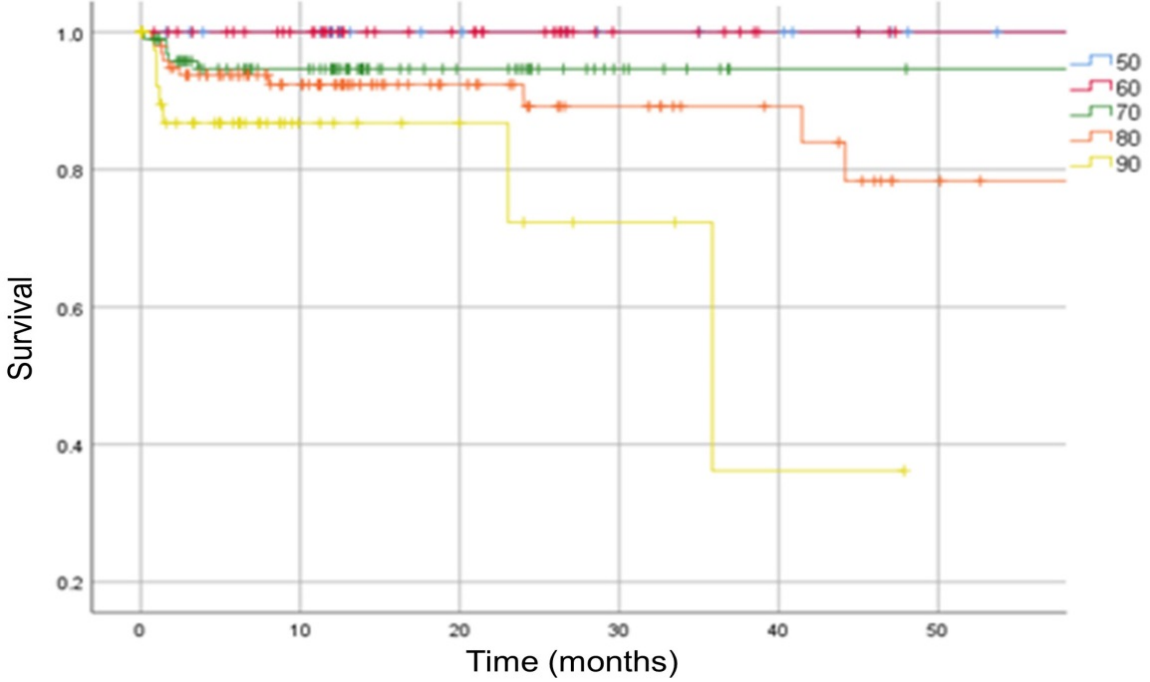
Kaplan-Meier survival curve. The analysis is separated in patients below 50 years old (blue 50 curve), then at 10-year intervals. Vertical marks indicate censoring.

Increasing age has also been correlated with a significantly higher mortality risk (p < 0.05). We report no mortality among patients below 60 years of age [Figure 4].

### Mortality depending on type of prosthesis

**Figure 5:**
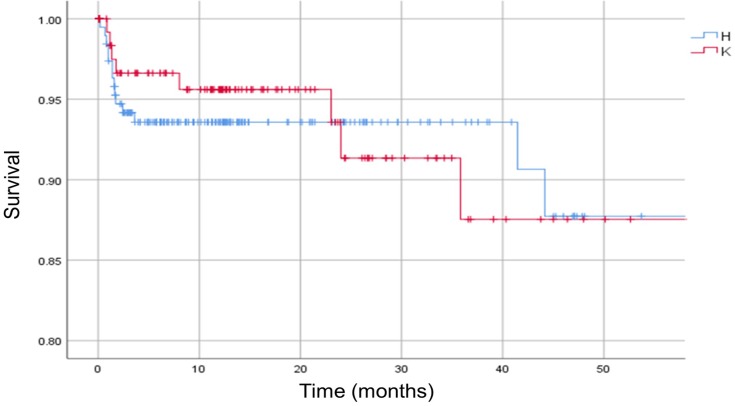
Kaplan-Meier survival curve. The analysis is separated in hip (H) and knee (K) infections. Vertical marks indicate censoring.

No significant difference in mortality was observed between patients with hip infections and those with knee infections (p > 0.05) [Figure 5].

### Mortality depending on infecting organism

**Figure 6:**
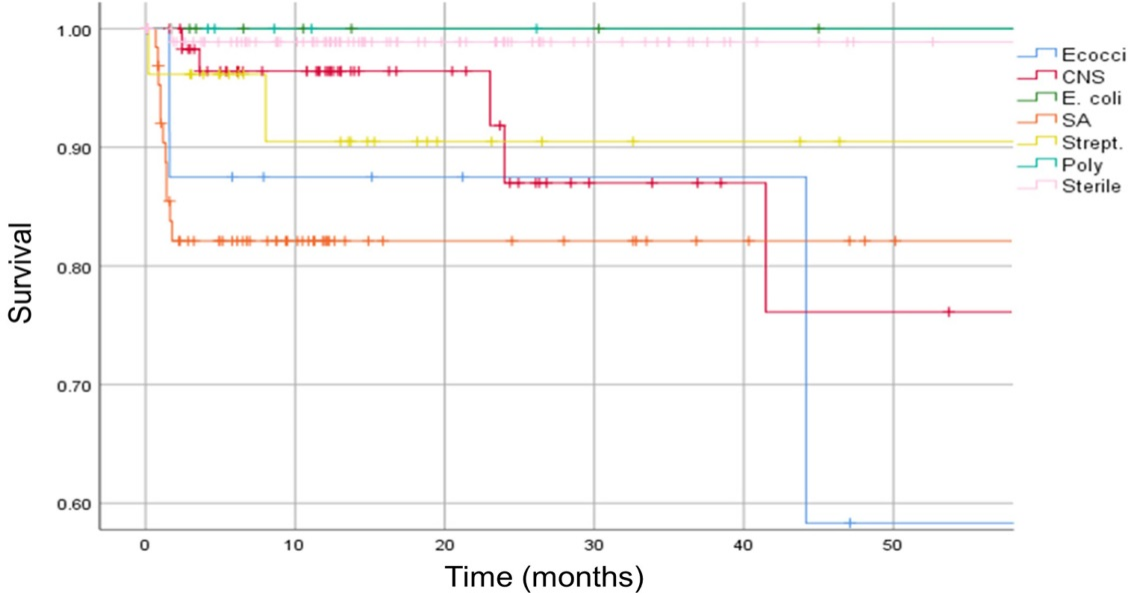
Kaplan-Meier survival curve. The analysis is separated in different infecting organisms. Vertical marks indicate censoring. Ecocci = enterococci, CNS = coagulase-negative staphylococci, E. coli = *Escherichia coli*, SA = *Staphylococcus aureus*, Strept. = *Streptococcus spp.*, Poly = polymicrobial

Patients with prosthetic-joint infection caused by enterococci or *methicillin-resistant Staphylococcus aureus* (MRSA) had a higher mortality risk than if the infection was caused by other bacteria species (p < 0.05, HR enterococci 2.27, 95% CI 0.53-9.65) [Figure 6].

### Mortality depending on type of surgical treatment

**Figure 7:**
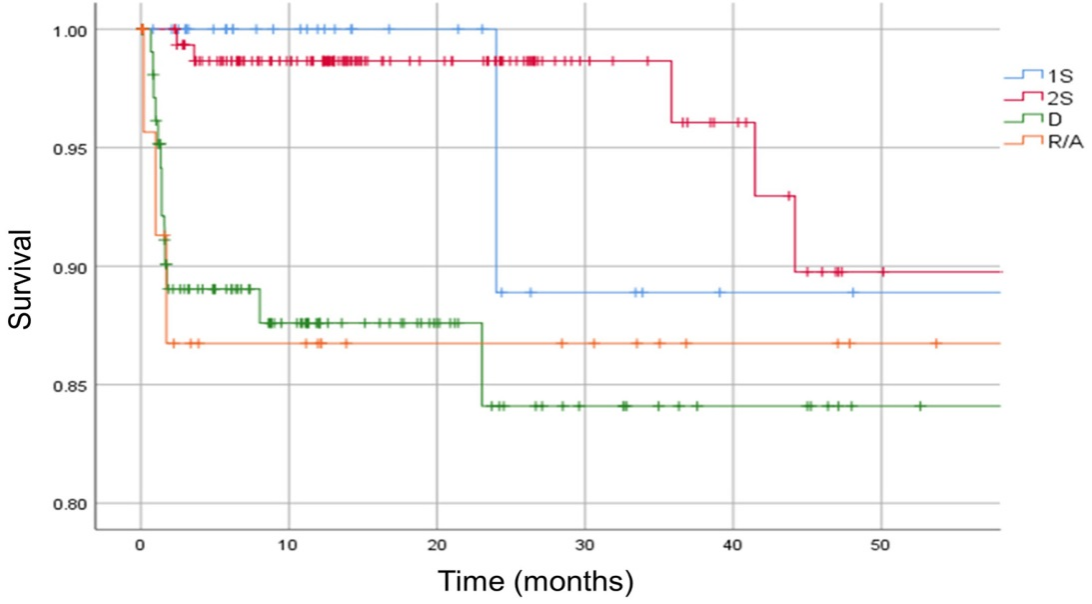
Kaplan-Meier survival curve. The analysis is separated in different types of surgical treatment. Vertical marks indicate censoring. 1S = one-stage exchange, 2S = two-stage exchange, D = debridement, R/A = removal or arthrodesis.

We found that patients treated with a one-stage or two-stage exchange had a significantly lower mortality than those treated with debridement and retention (p < 0.05, HR debridement 2.25, 95% CI 0.51-9.88) [Figure 7].

## Discussion

This study investigates the impact of prosthetic-joint infection on mortality. Our study shows a lower mortality at one year (5.5%) than the rate of 8% described by Overgaard et al. for total hip arthroplasty.^7^ Our results are closer to the recent ones by Lum et al.^3^,^9^,^10^

As already reported, we found no difference between hip and knee prosthesis but a higher mortality in men compared to women and an increased mortality with age.^3^,^4^,^7^,^11^-^13^ Higher mortality due to enterococci has already been hypothesized to be related to the intrinsic antimicrobial resistance to *b*-lactams which are used as prophylactic antibiotics before revision surgery.^7^ The current study also shows surprisingly, and never described before, that patients treated with a one-stage or two-stage exchange have a lower mortality than the ones treated with debridement and retention. We could have hypothesized that the two-stage reimplantation group would have a higher mortality given the increased number of operations and increased number of in-hospital days. The reason could be that patients with more comorbidities were not able to support more invasive treatments. These patients had a debridement and retention instead of the one or two-stage exchange needed. The fact that adhering to the algorithm prescribed by Werner Zimmerli was not feasible could result in a higher proportion of comorbid patients, and consequently a higher mortality, in the debridement and retention group.^2^ However, our conclusion is limited since our research was not designed to consider the patients' comorbidities and the cause of death. Therefore, more research is needed to understand the reason for this mortality and to investigate whether there are other patient selection criteria, such as comorbidities, that can aid in surgical selection. It would be useful to apply a more adapted algorithm which takes into account patients at risk of increased mortality.

Our study has other limitations. Its retrospective nature and reliance upon electronic files might underestimate the true incidence of death. Without using a Social Security death index, it is likely that the death of some patients was not captured in the database. Another major loss of patients was due to the inability to follow up those who came from a regional hospital and went back after the treatment. Nevertheless, using a Kaplan-Meier analysis, our observation that the mortality seems to be lower than anticipated is valid.^3^,^7^ Our conclusion is also limited due to the single-hospital perspective which undermine the external validity of our study.

## Conclusion

In summary, this study shows that the mortality is still high and differs according to sex, age, infecting organism and type of surgical treatment. Prosthetic-joint infections should be a concern to patients and clinicians. Indeed, before undergoing total hip or total knee arthroplasty, patients should be informed that prosthetic-joint infection is also associated with an increased risk of death. Finally, there is a need of studies to improve the management of patients at risk of increased mortality.

## Figures and Tables

**Table 1:**
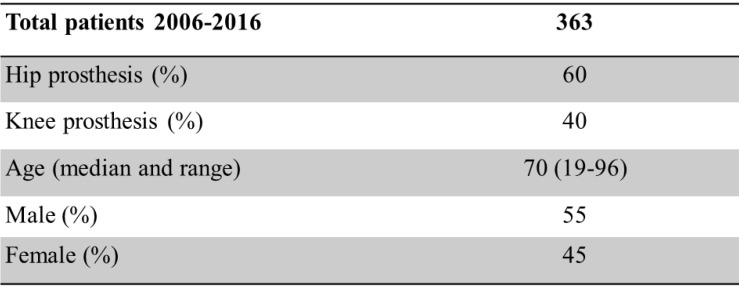
Patients' demographic characteristics.
